# Operationen bei benignen Schilddrüsenerkrankungen in Deutschland – Entwicklung von Fallzahl und Komplikationsraten im Verlauf von 10 Jahren

**DOI:** 10.1007/s00104-026-02459-4

**Published:** 2026-01-30

**Authors:** Martina T. Mogl, Henning Dralle, Claus Fahlenbrach, Christian Günster, Elke Jeschke, Dietmar Simon, Thomas Steinmüller, Eva Tusch, Matthias Maneck

**Affiliations:** 1 Chirurgische Klinik, Campus Charité Mitte, Campus Virchow-Klinikum, Charité-Universitätsmedizin Berlin, Corporate Member of Freie Universität Berlin, Humboldt-Universität zu Berlin, and Berlin Institute of Health, 10117 Berlin, Deutschland; 2 Klinik für Allgemein‑, Viszeralchirurgie, Sektion Endokrine Chirurgie und Schilddrüsen- und Nebenschilddrüsenchirurgie, Regio Klinikum Pinneberg, Pinneberg, Deutschland; 3 AOK-Bundesverband, Berlin, Deutschland; 4 Wissenschaftliches Institut der AOK, Rosenthaler Str. 31, 10178 Berlin, Deutschland; 5 Klinik für Allgemein- und Viszeralchirurgie, Thoraxchirurgie und Endokrine Chirurgie, Ev. Krankenhaus BETHESDA, Duisburg, Deutschland; 6 Klinik für Allgemein- und Viszeralchirurgie, DRK Kliniken Westend, Berlin, Deutschland; 7 Medizinischer Dienst der Krankenversicherung Berlin-Brandenburg, Berlin, Deutschland

**Keywords:** Schilddrüsenoperation, Stimmbandparese, Nachblutung, Fallzahl, Krankenhausvolumen, Thyroid gland operation, Vocal cord palsy, Postoperative bleeding, Case numbers, Hospital volume

## Abstract

**Hintergrund:**

Die Zahl der Schilddrüseneingriffe in Deutschland sank kontinuierlich über die letzten Jahre. Für die typischen Komplikationen Stimmbandparese, Hypokalziämie, Wundinfektion sowie Nachblutungen bestehen weiterhin unterschiedliche Inzidenzen in den verschiedenen Krankenhäusern.

**Fragestellung:**

Haben die Fallzahl oder die Krankenhauskategorie Einfluss auf die Komplikationsrate und haben sich in den letzten 10 Jahren die Verteilung der Operationen und die Komplikationsrate verändert?

**Material und Methoden:**

Die vorliegende retrospektive Beobachtungsstudie basiert auf anonymisierten Routinedaten der Allgemeinen Ortskrankenkasse (AOK) und wertet Schilddrüsenoperationen bei benigner Indikation zwischen 2011 und 2021 aus.

**Ergebnisse und Diskussion:**

Es zeigt sich eine Abnahme der Eingriffshäufigkeit bei benignen Schilddrüsenerkrankungen im 10-Jahres-Verlauf in Deutschland mit einer Verschiebung der Eingriffshäufigkeit hin zu fallzahlstarken Kliniken. Die Komplikationsraten für permanente Stimmbandparese und revisionsbedürftige Nachblutung sanken signifikant. Dieser Rückgang an Komplikationen konnte weder durch patient:innenspezifische Risikofaktoren noch durch die beobachtete Verschiebung der Fallverteilung erklärt werden.

**Graphic abstract:**

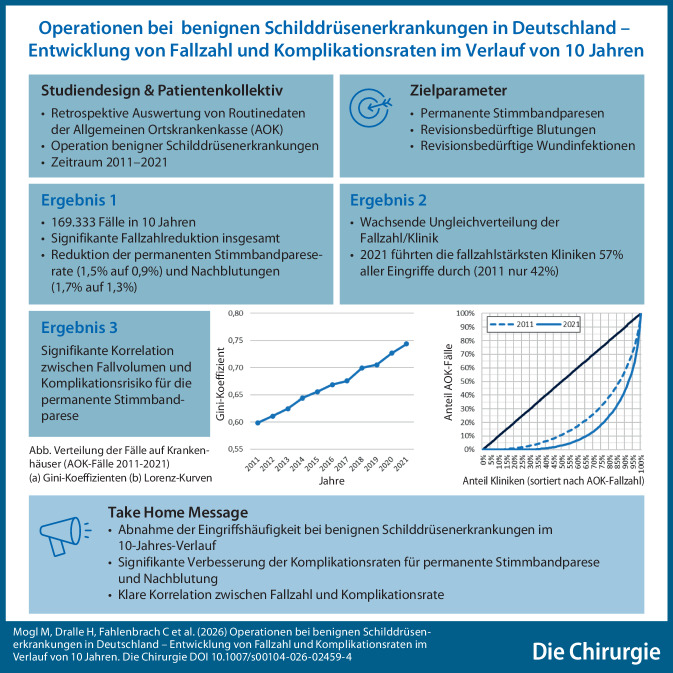

**Zusatzmaterial online:**

Die Online-Version dieses Artikels (10.1007/s00104-026-02459-4) enthält eine weitere Tabelle 5: Logistische Regressionsanalyse zur Bestimmung des Einflusses (Odds Ratio) der Zeit auf die Endpunkte unter Berücksichtigung weiterer Risikofaktoren (AOK-Fälle 2011–2021).

## Abstract (en)

**Background:**

The number of thyroid gland operations in Germany has dropped continuously over the past 10 years. The typical complications of vocal cord paralysis, hypocalcemia, wound infection and postoperative bleeding still occur with variable incidences in the different hospitals.

**Objective:**

Do the case numbers or hospital category have an influence on the complication rate and have the distribution of operations and the complication rates changed over the last 10 years?

**Material and methods:**

This retrospective observational study is based on anonymized routine data of the General Local Health Insurance Company (Allgemeine Ortskrankenkasse, AOK) and analyses data of thyroid gland operations for benign diseases between 2011 and 2021.

**Results and discussion:**

A continuous decrease in the frequency of operations for benign thyroid gland diseases over the course of 10 years in Germany could be confirmed. Additionally, a shift of operations towards hospitals with higher numbers of interventions could be demonstrated. The complication rates of permanent vocal cord paralysis and postoperative bleeding requiring revision have significantly declined. This reduction of complications could not be attributed to patient-specific risk factors or to the observed shift in the distribution of cases across hospitals.

## Hintergrund

Schilddrüsenoperationen gehören zu den häufigsten chirurgischen Eingriffen in Deutschland und werden in Krankenhäusern jeder Versorgungsstufe durchgeführt. Die Mehrheit der Indikationen betrifft benigne Erkrankungen bei Menschen jeder Altersgruppe [1]. Insgesamt sinkt die Zahl der Schilddrüsenoperationen in Deutschland kontinuierlich von fast 90.000 Eingriffen im Jahr 2011 auf knapp 63.000 Eingriffe im Jahr 2023 [1]. Die häufigsten Komplikationen sind Stimmbandparese, Hypokalziämie und Hypoparathyreoidismus sowie Wundinfektionen und revisionsbedürftige Nachblutungen.

Die Stimmbandparese bedeutet für die Betroffenen häufig eine Beeinträchtigung der Lebensqualität [2, 3], sie kann aber auch für die Operierenden medikolegale Konsequenzen haben [4]. Die Inzidenz der permanenten Stimmbandparese wird in der Literatur mit einer Schwankungsbreite von 0,86 %–6,6 % angegeben [5, 6] und ist neben patient:innenspezifischen Faktoren auch abhängig von der Erfahrung der Operierenden [7–9]. So ließ sich zeigen, dass bereits ab 20 Thyreoidektomien pro Operierenden und Jahr die Komplikationsrate sinkt [7].

Gleiches gilt für den postoperativen Hypoparathyreoidismus, der mit einer Inzidenz von 6,4 % für den passageren und 1,5 % für den permanenten Hypoparathyreoidismus beschrieben ist [10]. Angaben zur Inzidenz sind durch die in der Literatur unterschiedlichen Definitionen erschwert [11]. Auch für den Hypoparathyreoidismus ließ sich zeigen, dass ab einem jährlichen Volumen von 50 Eingriffen pro Jahr das Risiko von 6 % auf 3 % sinkt [12, 13].

Hierbei zeigen die Daten überwiegend den direkten Einfluss der Eingriffshäufigkeit pro Operateur und nachrangig das Fallvolumen pro Krankenhaus [9].

Etwas seltener treten Wundinfektionen und revisionspflichtige Nachblutungen nach Schilddrüseneingriffen auf mit 0,8 % bzw. 1,5 % pro Jahr [13, 14]. Die Datenlage zur Korrelation von Fallzahl pro Operateur und Nachblutungsrate ist uneindeutig [12, 15], allerdings scheint eine Rate von < 2 % bei 50 Eingriffen ein sinnvoller Qualitätsindikator zu sein [16].

Ob auch die Krankenhauskategorie einen Einfluss auf die Komplikationsrate hat, wird unterschiedlich diskutiert. Insgesamt besteht ein Zusammenhang zwischen Fallzahl und Komplikationsrate bei Schilddrüseneingriffen, und die zusammenfassende Analyse der ESES bestätigte die niedrigste Komplikationsrate von 6,1 % für Operateure mit 30 bis 100 Eingriffen pro Jahr im Vergleich zu einer Rate von 8,6 % für Chirurg:innen mit weniger als 10 Eingriffen pro Jahr [16–18].

Dies führte bereits zu Empfehlungen zur Konzentration von Schilddrüseneingriffen zugunsten der Sicherheit der Patientinnen und Patienten [16].

Nach den bereits früher publizierten Analysen aus AOK-Routinedaten [19, 20] stellt sich die Frage, ob über einen 10-Jahres-Zeitraum signifikante Veränderungen in der Verteilung der Schilddrüsenoperationen bei benigner Indikation in Deutschland und eine Veränderung der Komplikationsrate zu beobachten sind.

## Material und Methoden

### Datengrundlage

Die vorliegende retrospektive Beobachtungsstudie basiert auf anonymisierten Routinedaten der Allgemeinen Ortskrankenkasse (AOK). Erhoben wurden Krankenhaus- und vertragsärztliche Diagnosen und Prozeduren, Arzneimittelverordnungen sowie Versichertenstammdaten (Alter, Geschlecht, Überlebensstatus).

Eingeschlossen wurden Versicherte, bei denen zwischen 2011 und 2021 eine Thyreoidektomie, Hemithyreoidektomie, partielle bzw. subtotale Resektion oder Schilddrüsenoperation via Sternotomie (OPS 5‑061 bis 5‑064) durchgeführt und als Hauptdiagnose eine euthyreote Struma, Hyperthyreose oder Thyreoiditis (ICD-10: E01/E04–E06, D34) dokumentiert wurde. Ausgeschlossen wurden Patient:innen mit Alter unter 18 Jahren, mit Entfernung von zervikalen Lymphknoten (OPS: 5‑40[1/2/6].0, 5‑403), mit Krebserkrankungen (ICD-10: C00-C97, D00-D09, D37-D48) oder mit Hyperparathyreoidismus (ICD-10: E21.0-E21.3). Eingriffe bei maligner Schilddrüsenerkrankung wurden ausgeschlossen, da die Verschlüsselung von Prozedur und Diagnose keinen sicheren Rückschluss auf das jeweilige Erkrankungsstadium zulässt. Die Komplikationsraten sind somit nicht eindeutig nach Fallschwere zu stratifizieren.

### Krankenhausvolumen

Das jährliche Operationsvolumen je Klinik wurde anhand der im Strukturierten Qualitätsbericht (SQB) gemeldeten Schilddrüsenoperationen (OPS 5‑061 bis 5‑064) ermittelt [21]. Die AOK-Fälle jedes Jahres wurden in Quintile eingeteilt, wobei Quintil I die Kliniken mit dem niedrigsten und Quintil V jene mit dem höchsten Fallvolumen repräsentiert. Die Fallzahlgrenzen der Volumenkategorien wurden jährlich neu berechnet und der sinkenden Gesamtfallzahl angepasst. Für die Jahre 2011 und 2021 waren die Grenzen wie folgt: I: ≤ 63 bzw. ≤ 57; II: 64–116 bzw. 58–106; III: 117–210 bzw. 107–211; IV: 211–445 bzw. 212–474; V: ≥ 453 bzw. ≥ 475.

### Endpunkte

Als Endpunkte dienten die vom Wissenschaftlichen Institut der AOK definierten Qualitätsindikatoren bei benignen Schilddrüsenerkrankungen: permanente Stimmlippenlähmungen (181 bis 365 Tage), Logopädieverordnungen (≥ 21 Einheiten, 365 Tage) revisionsbedürftige Blutung (0 bis 7 Tage) und revisionsbedürftige Wundinfektion (3 bis 14 Tage) [19, 22, 23].

Permanente Stimmlippenlähmungen wurden anhand von ambulanten Daten über die Kombination von Lupenlaryngoskopie (EBM: 09311, 20310) und Lähmung der Stimmlippen (ICD-10: J38.0) durch denselben Arzt binnen 181 bis 365 Tagen nach Krankenhausentlassung abgebildet. Patient:innen mit präoperativ bestehender ambulanter Diagnose einer Stimmlippenlähmung wurden nicht gezählt.

Logopädieverordnungen wurden anhand von logopädischen Behandlungen bei organisch bzw. funktionell bedingten Erkrankungen der Stimme gemäß Heilmittelkatalog [24] abgebildet. Verordnungen wurden je Patient innerhalb von 365 Tagen summiert. Der Indikator zählt Patient:innen, bei denen die Gesamtverordnungsmenge des Regelfalls (20 Einheiten) überschritten wurde. Patientinnen und Patienten mit präoperativen Logopädieverordnungen wurden nicht gezählt.

Revisionsbedürftige Blutungen am Operationstag oder innerhalb von 7 Tagen nach der Operation wurden über die Kombination der Blutungsdiagnose (ICD-10: T81.0) und einer der Prozeduren Einlegen einer Drainage, Revision der Operationswunde oder Reoperation (OPS: 5‑060.[1,3], 5‑983) abgebildet.

Revisionsbedürftige Wundinfektionen innerhalb von 3 bis 14 Tagen nach der Operation wurden über die Kombination der Diagnosen Infektion oder Sepsis (ICD-10: A4[0,1], B9[5,6], T81.4) und einer der oben genannten Prozeduren (OPS: 5‑060.[1,3], 5‑983) abgebildet. Zusätzlich wurde Wunddébridement am Hals (OPS: 5‑896.[0,1,2,3,x]5) als revisionsbedürftige Wundinfektion gezählt.

### Statistische Analyse

Deskriptive Kennzahlen wurden für das Gesamtkollektiv und die Einzeljahre berechnet. Trends wurden mittels linearer Regression bei α = 0,05 und einer Bonferroni-Korrektur (Faktor 34) geprüft. Für die Endpunkte erfolgte ebenfalls eine Trendanalyse per linearer Regression.

Zur Abschätzung des Zeittrends auf die Indikatoren wurden multiple logistische Regressionsmodelle verwendet. Neben einer kontinuierlichen Jahresvariable wurde adjustiert nach Alter (Quintile), Geschlecht, Elixhauser-Begleiterkrankungen [25], Operationsumfang, intraoperativem Neuromonitoring (IONM), Rezidivstatus, Behandlungsanlass und präoperativer Medikation. Die Modellselektion erfolgte anhand des *Stepwise-backward*-Algorithmus (*p* > 0,04). Anschließend wurden die kontinuierliche Zeitvariable sowie das jährliche Fallvolumen integriert.

Die Entwicklung der Fallverteilung wurde anhand von Gini-Koeffizienten und Lorenz-Kurven für die einzelnen Jahre untersucht.

Die angegebenen Kennzahlen beziehen sich auf die jeweils auswertbare Fallmenge. Patient:innen, welche nicht vollständig nachbeobachtbar waren und bei denen kein Komplikationsereignis innerhalb des Nachbeobachtungszeitraums auftrat, wurden zensiert.

Alle Auswertungen wurden mit der Software STATA18.0 (StataCorp LLC, 4905 Lakeway Drive, College Station, TX 77845 USA) durchgeführt.

## Ergebnisse

Die deskriptive Analyse der 169.333 Fälle über den 10-Jahres-Zeitraum ergibt eine signifikante Reduktion der jährlichen Fallzahl von 18.360 im Jahr 2011 auf 11.535 im Jahr 2021. Deskriptive Statistiken des Fallkollektivs sind in Tab. 1 dargestellt.

**Tab. 1:** Deskriptive Statistiken der einbezogenen AOK-Fälle (2011–2021)

	2011	2012	2013	2014	2015	2016	2017	2018	2019	2020	2021	Gesamt	*p*^a^
Fallzahl	18.360	19.000	17.495	16.695	15.771	15.774	15.224	13.749	13.761	11.969	11.535	169.333	**<** **0,001**
Alter (Median [IQR])	54 (45–65)	54 (45–64)	54 (44–64)	53 (45–64)	54 (44–64)	53 (44–64)	53 (44–63)	53 (44–63)	54 (44–63)	53 (43–62)	54 (43–62)	54 (44–64)	1,000
Geschlecht (weiblich; %)	76,4	75,5	75,6	76,3	76,6	76,7	76,0	76,2	77,0	76,5	76,8	76,3	1,000
*Behandlungsanlass (%)*													
Euthyreote Struma	86,8	86,4	85,6	85,5	85,9	85,0	84,9	84,7	84,8	84,2	83,7	85,4	**<** **0,001**
Hyperthyreose	11,7	12,2	12,8	12,7	12,6	13,2	13,6	13,7	13,6	14,1	14,4	13,0	**<** **0,001**
Thyreoiditis	1,4	1,4	1,6	1,8	1,6	1,9	1,5	1,6	1,6	1,7	1,8	1,6	1,000
*Operationsverfahren (%)*													
Thyreoidektomie	54,1	55,1	55,5	55,6	55,0	55,1	53,9	52,8	52,2	51,7	51,0	54,0	**0,048**
Hemithyreoidektomie	21,1	23,3	25,2	26,2	28,2	29,5	31,1	33,0	33,6	35,1	36,3	28,6	**<** **0,001**
Subtotale Resektion	21,2	18,2	15,6	14,4	12,6	10,9	10,5	9,1	8,8	7,5	6,8	13,0	**<** **0,001**
Partielle Resektion	3,8	3,7	3,9	3,9	4,3	4,6	4,7	5,2	5,6	5,8	6,0	4,6	**<** **0,001**
*Präoperative Medikation (%)*													
Antithrombotika	6,4	6,6	7,0	7,2	7,1	7,0	7,1	7,6	7,1	7,4	7,7	7,1	**0,026**
Thyreostatika	14,1	14,4	15,0	15,0	14,3	15,2	14,7	15,5	15,1	15,2	14,9	14,8	1,000
Immunsuppressiva	0,6	0,5	1,0	1,2	1,1	1,1	1,2	1,4	1,3	1,4	1,8	1,1	**0,002**
Systemische Glukokortikoide	2,1	2,2	2,3	2,3	2,4	2,4	2,5	2,6	2,5	2,3	2,4	2,3	1,000
*Weitere Charakteristika (%)*													
Neuromonitoring	83,8	87,3	88,4	87,7	88,9	90,9	90,6	94,0	94,3	94,9	95,1	90,0	**<** **0,001**
Beidseitige Operation	75,2	73,4	71,7	70,9	68,9	67,6	66,0	64,2	63,3	61,3	60,2	68,2	**<** **0,001**
Mit Parathyreoidektomie	5,6	6,1	6,5	6,9	7,0	6,2	6,5	6,6	6,4	6,7	7,0	6,5	1,000
Reexploration	2,6	2,5	2,2	2,2	2,3	2,3	2,3	2,3	2,3	2,6	2,2	2,4	1,000
Sternotomie	0,3	0,3	0,2	0,3	0,2	0,3	0,2	0,3	0,3	0,3	0,3	0,3	1,000
Schild‑/Nebenschilddrüsenoperation im Vorjahr	0,4	0,5	0,5	0,5	0,7	0,6	0,8	0,6	0,7	0,8	0,7	0,6	0,061
Mehrknotige Struma	68,7	68,0	66,5	66,2	66,2	64,5	63,9	61,1	61,5	60,0	58,6	64,6	**<** **0,001**
Solitärer Schilddrüsenknoten	13,1	13,7	14,4	14,8	15,1	15,9	16,8	17,6	17,7	19,1	19,9	15,9	**<** **0,001**
Basedow-Krankheit^b^	5,2	5,6	6,3	6,5	6,6	7,1	8,1	8,8	8,6	9,2	9,5	7,2	**<** **0,001**
*Elixhauser-Begleiterkrankungen*^*c*^* (%)*													
Bluthochdruck	33,6	34,8	34,6	35,1	34,5	35,4	35,6	35,1	36,2	35,0	34,6	34,9	0,842
Adipositas	8,6	8,8	9,5	10,5	11,1	11,2	11,5	12,2	12,7	12,1	12,2	10,8	**<** **0,001**
Diabetes	9,9	9,8	9,9	10,2	10,4	10,3	9,8	9,3	9,7	9,2	9,3	9,8	0,750
Chronische Lungenerkrankung	4,6	5,0	4,9	5,2	5,6	5,3	5,7	5,9	5,8	5,7	5,9	5,3	**0,003**
Kardiale Arrhythmie	4,4	4,3	4,4	4,7	4,7	4,7	4,5	4,5	4,7	4,9	4,4	4,5	1,000
Störungen des Flüssigkeits‑/Elektrolythaushalts	3,2	3,4	3,6	3,5	3,4	3,6	3,2	3,0	3,2	3,2	3,2	3,3	1,000
Depression	2,6	2,6	2,9	2,9	3,2	3,4	3,3	3,1	3,2	3,1	3,4	3,0	0,158
Kongestive Herzerkrankung	1,9	2,0	2,0	2,0	2,0	1,9	1,7	1,8	1,7	1,7	1,6	1,9	**0,038**
Nierenversagen/-insuffizienz	1,3	1,6	1,6	2,0	2,1	2,1	2,0	2,0	2,0	1,9	2,0	1,9	0,738
Weitere neurologische Erkrankungen	1,1	1,0	1,0	1,1	1,0	1,2	1,1	1,1	1,3	1,3	1,2	1,1	**0,006**
Periphere Gefäßerkrankung	0,9	0,9	1,0	1,0	1,0	1,1	1,0	1,2	1,0	1,0	0,9	1,0	1,000

^a^ *p*-Wert adjustiert für multiples Testen nach Bonferroni (Faktor 34)

^b^ ICD-10 E05.0 Hyperthyreose mit diffuser Struma (Inklusion: Basedow-Krankheit, toxische diffuse Struma, toxische Struma ohne nähere Angabe)

^c^ Komorbiditäten mit einer Gesamthäufigkeit von unter 1 % werden nicht dargestellt (Erkrankung der Herzklappen, Koagulopathie, rheumatische Erkrankung, Lebererkrankung, Psychosen, Lähmung, Defizienzanämie, pulmonale Herzkrankheit und Krankheiten des Lungenkreislaufes, Gewichtsverlust, Alkoholabusus, Drogenabusus, Blutungsanämie, peptisches Ulkus ohne Blutung, AIDS/HIV)

Im Indikationsspektrum zeigt sich eine signifikante Abnahme der euthyreoten Struma von 86,8 % im Jahr 2011 auf 83,7 % im Jahr 2021 (*p* < 0,001) und eine Zunahme der Fälle mit Hyperthyreose von 11,7 % auf 14,4 % (*p* < 0,001), während sich der Anteil der Eingriffe bei Thyreoiditis insgesamt nicht signifikant veränderte (von 1,4 % auf 1,8 %). Die Betrachtung der Operationsverfahren zeigt eine Reduktion der Thyreoidektomien und subtotalen Resektionen im 10-Jahres-Verlauf (von 54,1 %, bzw. 21,2 % auf 51,0 %, bzw. 6,8 %), während der Anteil der Hemithyreoidektomien signifikant zunahm (21,1 % auf 36,3 %, *p* < 0,001). Eine genauere Analyse des Indikationsspektrums zeigt eine signifikante Zunahme der Operationen bei Hyperthyreose mit diffuser Struma (inklusive Morbus Basedow) von 5,2 % auf 9,5 % sowie solitärer Schilddrüsenknoten (jeweils *p* < 0,001), während der Anteil an Reexplorationen und Sternotomien konstant blieb.

Es lässt sich eine signifikante Steigerung für die Verwendung des Neuromonitorings (83,8 % auf 95,1 %) demonstrieren und eine Abnahme beidseitiger Eingriffe (75,2 % auf 60,2 %, *p* < 0,001).

Hinsichtlich patient:innenspezifischer Charakteristika offenbart sich eine Zunahme der Anwendung von Antithrombotika (6,4 % auf 7,7 %) und Immunsuppressiva (0,6 % auf 1,8 %) im 10-Jahres-Verlauf, während die Anwendung thyreostatischer Medikation und Glukokortikoide keine signifikanten Veränderungen zeigten. Weiterhin manifestiert sich eine signifikante Zunahme der Begleiterkrankungen Adipositas (8,6 % auf 12,2 %, *p* > 0,001) und chronische Lungenerkrankungen (4,6 % auf 5,9 %, *p* = 0,003). Eine signifikante Reduktion stellte sich für kongestive Herzerkrankungen dar (1,9 % auf 1,6 %, *p* = 0,038) (Tab. 1).

Die Entwicklung der Indikatorhäufigkeiten ist in Tab. 2 dargestellt. Im 10-Jahres-Verlauf sanken die Häufigkeiten für permanente Stimmbandlähmungen und revisionsbedürftige Blutungen von 1,5 % (2011) auf 0,9 % (2021) bzw. von 1,7 % (2011) auf 1,3 % (2021). Die Häufigkeiten von Logopädieverordnungen (≥ 21 Einheiten) und revisionsbedürftigen Wundinfektionen blieben mit 0,7 % bzw. 0,3 % nahezu konstant. Unter Berücksichtigung von Risikofaktoren wie Alter, Geschlecht, Begleiterkrankungen sowie spezifischen Faktoren sank das Risiko für eine permanente Stimmbandlähmung um etwa 3 % pro Jahr (OR [95 %-KI]: 0,97 [0,95–0,98]). Für revisionsbedürftige Blutungen sank das jährliche Risiko um etwa 4 % (OR [95 %-KI]: 0,96 [0,95–0,98]). Die logistischen Regressionsmodelle sind in Tab. 5 (Zusatzmaterial online) dargestellt. Für Logopädieverordnungen und revisionsbedürftige Wundinfektionen wurde keine signifikante Verringerung des Risikos beobachtet.

**Tab. 2:** Indikatorhäufigkeiten sowie Odds Ratios (OR) des Zeitparameters (Jahre) der multivariaten logistischen Regression (AOK-Fälle 2011–2021)

Jahr	Permanente Stimmbandlähmung (%)	Logopädie (≥ 21 Einheiten) (%)	Revisionsbedürftige Blutung (%)	Revisionsbedürftige Wundinfektion (%)
2011	1,5	0,7	1,7	0,3
2012	1,4	0,7	1,9	0,3
2013	1,4	0,8	1,9	0,4
2014	1,3	0,6	1,6	0,3
2015	1,2	0,7	1,8	0,4
2016	1,3	0,7	1,4	0,3
2017	1,4	0,8	1,4	0,3
2018	1,2	0,7	1,4	0,3
2019	1,2	0,6	1,4	0,3
2020	1,0	0,6	1,2	0,2
2021	0,9	0,6	1,3	0,3
Gesamt	1,3	0,7	1,6	0,3
*Odds Ratio *Jahre	0,97 (0,95–0,98)	0,98 (0,97–1,01)	0,96 (0,95–0,98)	0,97 (0,94–1,01)

**Abb. 1:**
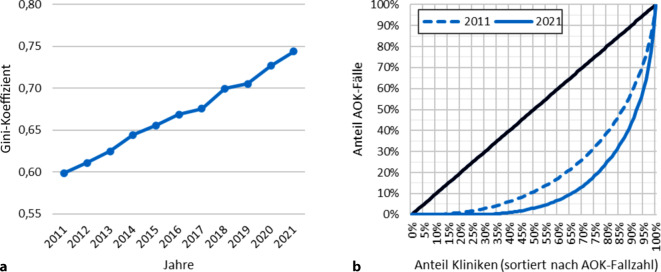
Verteilung der Fälle auf die Krankenhäuserhäuser (AOK-Fälle 2011–2021). (**a**) Gini-Koeffizienten. (**b**) Lorenz-Kurven

Ob die abnehmende Fallzahl mit einer Veränderung der Fallverteilung über die 1077 Kliniken einherging, wurde anhand von Gini-Koeffizienten überprüft. Diese stiegen im 10-Jahres-Verlauf stetig von 0,60 in 2011 auf 0,74 in 2021 (Abb. 1a). Dieser Anstieg beschreibt eine wachsende Ungleichverteilung der Fälle auf die Kliniken. In Abb. 1b sind die Fallverteilungen für die Jahre 2011 und 2021 exemplarisch mittels Lorenz-Kurven dargestellt. Sie machen deutlich, dass in den jeweils 50 % der fallzahlschwächsten Kliniken (*n* = 539) der Anteil an Operationen von 10 % in 2011 auf 3 % in 2021 sank. Demgegenüber fanden in den jeweils 10 % der fallzahlstärksten Kliniken (*n* = 108) in 2011 42 % aller Operationen und 2021 57 % aller Operationen statt. Die Fallzahl nahm in den fallzahlschwächeren Kliniken überproportional ab.

Um zu überprüfen, ob die Veränderung der Fallverteilung Einfluss auf das Absinken des Risikos für eine permanente Stimmbandlähmung oder revisionsbedürftige Blutung hatte, wurden die logistischen Regressionsmodelle um Parameter für das Fallvolumen erweitert (Tab. 3). Der Einfluss des Zeitparameters auf die Indikatoren bleibt unverändert. Das Risiko für eine permanente Stimmbandlähmung bzw. revisionsbedürftige Blutung sinkt weiterhin um etwa 3 % bzw. 4 % pro Jahr (OR [95 %-KI]: 0,97 [0,95–0,98] bzw. 0,96 [0,95–0,98]). Weitere signifikante Risikofaktoren waren für permanente Stimmbandlähmungen z. B. Rezidivoperation (OR 2,47 [2,04–2,98]) und Alter ≥ 67 Jahre (OR 2,34 [2,02–2,70]). Das Vorliegen eines solitären Schilddrüsenknotens zeigte ein signifikant reduziertes Risiko für die permanente Stimmbandparese (0,53 [0,45–0,63]). Bei revisionsbedürftigen Blutungen hatten z. B. das Vorliegen einer Koagulopathie (OR 4,05 [3,27–5,03]), Störungen des Wasser- und Elektrolythaushalts sowie des Säure-Basen-Gleichgewichts (OR 2,73 [2,28–3,28]) oder Alkoholabusus (OR 2,21 [1,3–3,77]) ebenfalls signifikanten Einfluss.

**Tab. 3:** Logistische Regressionsanalyse zur Bestimmung des Einflusses (Odds Ratio) der Zeit auf die Endpunkte unter Berücksichtigung des jährlichen Fallvolumens je Krankenhaus und weiterer Risikofaktoren (AOK-Fälle 2011–2021)

	Permanente Stimmbandlähmung	Revisionsbedürftige Blutung
*Zeit in Jahren*	0,97 (0,95–0,98)	0,96 (0,95–0,98)
*Jährliches Fallvolumen (SQB)*^*a*^		
VK 1	1,88 (1,47–2,42)	0,80 (0,68–0,94)
VK 2	1,63 (1,26–2,11)	0,82 (0,70–0,97)
VK 3	1,48 (1,13–1,92)	0,89 (0,75–1,06)
VK 4	1,18 (0,90–1,56)	0,85 (0,71–1,00)
VK 5	1 (Referenz)	1 (Referenz)
*Alter in Jahren*		
18–42	1 (Referenz)	1 (Referenz)
43–50	1,25 (1,06–1,48)	1,26 (1,08–1,46)
51–57	1,46 (1,26–1,70)	1,64 (1,43–1,89)
58–66	1,84 (1,58–2,15)	1,75 (1,52–2,02)
≥ 67	2,34 (2,02–2,70)	1,68 (1,45–1,95)
*Geschlecht (w)*	1,23 (1,1–1,37)	0,73 (0,67–0,8)
*Operationsverfahren und Morphologie*		
Reexploration	2,47 (2,04–2,98)	1,36 (1,07–1,72)
Beidseitige Operation	0,69 (0,62–0,77)	1,20 (1,08–1,32)
Mehrknotige Struma	–	1,26 (1,13–1,40)
Solitärer Schilddrüsenknoten	0,53 (0,45–0,63)	–
Thyreoiditis	1,47 (1,09–1,99)	–
Basedow-Krankheit^b^	–	1,48 (1,23–1,79)
Neuromonitoring	0,95 (0,81–1,11)	–
*Präoperative Medikation*		
Systemische Glukokortikoide	–	0,74 (0,58–0,95)
Thyreostatika	–	1,37 (1,23–1,54)
*Begleiterkrankungen*		
Störungen des Flüssigkeits- und Elektrolythaushalts	1,62 (1,33–1,98)	2,73 (2,28–3,28)
Kardiale Arrhythmie	–	1,63 (1,39–1,90)
Koagulopathie	–	4,05 (3,27–5,03)
Adipositas	–	0,83 (0,74–0,94)
Alkoholabusus	–	2,21 (1,30–3,77)
Bluthochdruck, ohne Komplikationen	–	1,17 (1,07–1,27)
Weitere neurologische Erkrankungen	–	1,40 (1,05–1,87)
Chronische Lungenerkrankung	1,35 (1,15–1,59)	–
Lähmung	1,76 (1,01–3,07)	–

Mit „–“ gekennzeichnete Risikofaktoren waren aufgrund fehlender Signifikanz nicht in der Risikoadjustierung enthalten

^a^ Die Fallzahlgrenzen der Volumenkategorien wurden jährlich neu berechnet. Für die Jahre 2011 und 2021 waren die Grenzen wie folgt: VK I: ≤ 63 bzw. ≤ 57; VK II: 64–116 bzw. 58–106; VK III: 117–210 bzw. 107–211; VK IV: 211–445 bzw. 212–474; VK V: ≥ 453 bzw. ≥ 475

^b^ ICD-10 E05.0 Hyperthyreose mit diffuser Struma (Inklusion: Basedow-Krankheit, toxische diffuse Struma, toxische Struma ohne nähere Angabe)

**Abb. 2:**
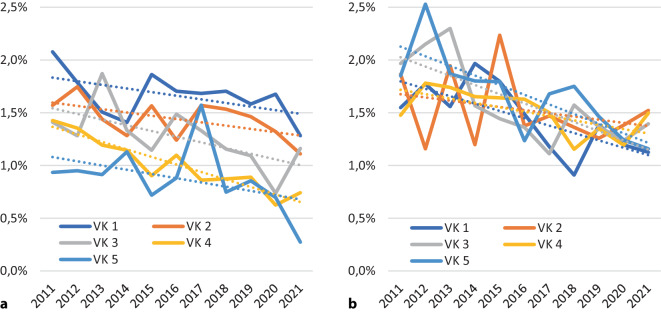
Jährliche Indikatorhäufigkeit nach Volumenkategorien (VK; AOK-Fälle 2011–2021) für (**a**) permanente Stimmbandlähmungen und (**b**) revisionsbedürftige Blutungen

Die Operation bei Basedow-Erkrankung oder anderen Hyperthyreosen hat ebenfalls ein erhöhtes Risiko für eine revisionsbedürftige Blutung (OR 1,48 [1,23–1,79]) sowie die präoperative Einnahme von Thyreostatika (OR 1,37 [1,23–1,54]) (Tab. 3). Die Modelle zeigten zusätzlich mit abnehmendem Fallvolumen ein steigendes Risiko für permanente Stimmbandlähmungen und ein abnehmendes Risiko für revisionsbedürftige Blutungen. Die Abb. 2a, b zeigen die Entwicklung der Indikatorhäufigkeiten für permanente Stimmbandlähmungen und revisionsbedürftige Blutungen für die einzelnen Volumenkategorien. Bei beiden Indikatoren sinken die Häufigkeiten in allen Volumenkategorien. Die Graphen deuten darauf hin, dass im 10-Jahres-Verlauf Unterschiede zwischen den Kategorien bei permanenten Stimmbandlähmungen bestehen bleiben, während sie bei den revisionsbedürftigen Blutungen abnehmen.

Das Bestehen eines Volume-Outcome-Zusammenhangs wurde anhand des aktuellen 3‑Jahres-Zeitraums (2019–2021) überprüft. Die Ergebnisse der Regressionsanalyse sind in Tab. 4 dargestellt. Bei den permanenten Stimmbandlähmungen zeigte sich ein signifikanter Einfluss des Fallvolumens. Mit sinkendem Fallvolumen stieg das Komplikationsrisiko. Für revisionsbedürftige Blutungen zeigte sich kein signifikanter Zusammenhang zwischen Fallvolumen und Komplikationsrisiko. Weitere signifikante Einflussfaktoren bei permanenter Stimmbandlähmung waren erneut z. B. Rezidivoperation (OR 2,39 [1,49–3,82]), Alter ≥ 67 Jahre (OR 2,09 [1,58–2,77]) sowie das Vorliegen eines solitären Schilddrüsenknotens (0,57 [0,40–0,80]). Bei revisionsbedürftigen Blutungen hatte z. B. das Vorliegen einer Koagulopathie (OR 4,02 [2,41–6,73]) oder Störungen des Wasser- und Elektrolythaushalts sowie des Säure-Basen-Gleichgewichts (OR 2,62 [1,80–3,81]) einen signifikanten Einfluss.

**Tab. 4:** Logistische Regressionsanalyse zur Bestimmung des Einflusses (Odds Ratio) des jährlichen Fallvolumens je Krankenhaus auf die Endpunkte unter Berücksichtigung weiterer Risikofaktoren (AOK-Fälle 2019–2021)

	Permanente Stimmbandlähmung	Revisionsbedürftige Blutung
*Jährliches Fallvolumen (SQB)*^*a*^		
VK 1	**2,42 (1,68–3,47)**	0,91 (0,65–1,29)
VK 2	**2,10 (1,43–3,08)**	1,01 (0,71–1,43)
VK 3	**1,62 (1,07–2,45)**	0,99 (0,69–1,41)
VK 4	1,22 (0,85–1,75)	1,01 (0,67–1,51)
VK 5	1 (Referenz)	1 (Referenz)
*Alter in Jahren*		
18–42	1 (Referenz)	1 (Referenz)
43–50	–	1,54 (1,1–2,14)
51–57	1,57 (1,20–2,06)	2,06 (1,5–2,84)
58–66	1,70 (1,27–2,28)	1,92 (1,38–2,69)
≥ 67	2,09 (1,58–2,77)	2,11 (1,48–3,01)
*Geschlecht (w)*	1,52 (1,17–1,98)	0,76 (0,64–0,92)
*Operationsverfahren und Morphologie*		
Reexploration	2,39 (1,49–3,82)	1,62 (1,09–2,40)
Beidseitige Operation	0,67 (0,53–0,85)	–
Mehrknotige Struma	–	1,37 (1,12–1,69)
Solitärer Schilddrüsenknoten	0,57 (0,40–0,80)	–
Basedow-Krankheit^b^	0,59 (0,36–0,97)	2,07 (1,49–2,88)
*Präoperative Medikation*		
Thyreostatika	1,55 (1,1–2,17)	–
*Begleiterkrankungen*		
Störungen des Flüssigkeits- und Elektrolythaushalts	1,65 (1,07–2,54)	2,62 (1,8–3,81)
Adipositas	–	0,73 (0,55–0,96)
Koagulopathie	–	4,02 (2,41–6,73)
Kardiale Arrhythmie	–	1,93 (1,39–2,67)
Bluthochdruck, ohne Komplikationen	–	1,25 (1,03–1,52)
Gewichtsverlust	–	2,63 (1,10–6,26)
Kongestive Herzerkrankung	–	0,43 (0,21–0,89)
Pulmonale Herzkrankheit und Krankheiten des Lungenkreislaufes	3,50 (1,41–8,66)	–

Mit „–“ gekennzeichnete Risikofaktoren waren aufgrund fehlender Signifikanz nicht in der Risikoadjustierung enthalten

^a^ Die Fallzahlgrenzen der Volumenkategorien wurden jährlich neu berechnet. Für die Jahre 2019 und 2021 waren die Grenzen wie folgt: VK I: ≤ 54 bzw. ≤ 57; VK II: 55–111 bzw. 58–106; VK III: 112–203 bzw. 107–211; VK IV: 204–477 bzw. 212–474; VK V: ≥ 478 bzw. ≥ 475

^b^ ICD-10 E05.0 Hyperthyreose mit diffuser Struma (Inklusion: Basedow-Krankheit, toxische diffuse Struma, toxische Struma ohne nähere Angabe)

## Diskussion

Die Analyse der Operationen bei benigner Schilddrüsenerkrankung in Deutschland zeigt einige interessante Entwicklungen im 10-Jahres-Verlauf. Anhand der 169.333 AOK-Fälle manifestiert sich insgesamt ein Rückgang der Operationszahlen bei gleichzeitiger Verschiebung der Fälle in fallzahlstarke Kliniken.

Dies bestätigt den Trend der vorhergehenden Publikation und unterstreicht die Bedeutung der sich verändernden Kliniklandschaft Deutschlands [20, 26]. Die Reduktion der Eingriffe bei benignen Schilddrüsenerkrankungen weist auf eine strengere Indikationsstellung hin, wie in der deutschen S2k-Leitlinie empfohlen [27].

Die Betrachtung patient:innenspezifischer Faktoren zeigte eine Zunahme von Begleiterkrankungen wie Adipositas, kongestiver Herzerkrankungen und chronischer Lungenerkrankungen sowie einen signifikant steigenden Anteil von Patientinnen und Patienten mit Antithrombotika und Immunsuppressiva im 10-Jahres-Verlauf. Diese Faktoren werden teilweise als Risikofaktoren für Komplikationen nach operativen Eingriffen diskutiert [28]. Diese Faktoren zeigten auch in den hier verwendeten Modellen zum Teil signifikante Einflüsse.

Die vorliegende Studie zeigte im Langzeitverlauf signifikant sinkende Komplikationsraten für permanente Stimmbandlähmungen und revisionsbedürftige Blutungen. Dabei zeigte sich eine gute Übereinstimmung der Häufigkeiten mit Registerdaten. So berichteten Bartsch et al. auf Basis des StuDoQ-Registers eine Rate an permanenten Stimmbandlähmungen von 1,3 % für die Jahre 2017 und 2018 [32]. Für revisionsbedürftige Blutungen berichteten die Autoren im selben Zeitraum eine Rate von 1,4 %.

Im Zusammenhang mit revisionsbedürftigen Blutungen erscheint die Zunahme der Operationen bei Hyperthyreosen, insbesondere bei diffuser Struma, die auch Morbus Basedow umfasst, auffällig. Welche Ursache dieser Anstieg hat, lässt sich anhand der vorliegenden Daten kaum eruieren. Die medikamentöse Behandlung der Hyperthyreose stellt weiterhin die Erstlinientherapie dar. Allerdings zeigt eine kürzlich publizierte Langzeitanalyse von Verissimo et al., dass auch nach mehrjähriger medikamentöser Behandlung einer Hyperthyreose bei Morbus Basedow oder hyperthyreoter Knotenstruma noch Rezidive auftreten und nur die Chirurgie eine bis zu 100 %ige Heilung verspricht im Gegensatz zu Thyreostatika und Radiojodtherapie mit 41,7 % bzw. 82,5 % Remission [29]. Dies unterstreicht die Bedeutung der Thyreoidektomie als valide Behandlung der Hyperthyreose. Umso wichtiger erscheint die Analyse der Komplikationsraten nach operativer Therapie.

So zeigte eine Studie von Mohtashami et al. 2022, dass die Operation bei Basedow-Erkrankung mit einem signifikant erhöhten Risiko für postoperative Hämatome und Hypokalzämie assoziiert ist bei gleicher Recurrenspareserate [30]. Auch die hier untersuchte Kohorte zeigt ein erhöhtes Risiko für Nachblutungen bei Operationen wegen Morbus Basedow und hyperthyreoter Struma.

Auch in der oben genannten Studie von Bartsch et al. zeigte sich ein erhöhtes Risiko für Nachblutungen bei Basedow-Patient:innen [32].

Besonders war außerdem die steigende Zahl von Patientinnen und Patienten mit Antithrombotika, wobei v. a. die Assoziation zu Komplikationsraten interessant erscheint. Die Inzidenz von Hämatomen nach Thyreoidektomie wurde kürzlich in einer europäischen Erhebung mit 5,7 % bestimmt [31]. Es zeigte sich in 9 fallzahlstarken Kliniken kein Unterschied zwischen Patientinnen und Patienten mit oraler Koagulation gegenüber nicht antikoagulierten Betroffenen, wobei nur etwa ein Viertel der Fälle (1,27 %) eine operative Revision benötigte. Das Risiko einer revisionsbedürftigen Nachblutung war auch in unserer Kohorte bei Vorliegen einer Koagulopathie signifikant erhöht. Allerdings erlaubt die Datenanalyse nicht die Unterscheidung der zugrunde liegenden Koagulopathie und auch nicht der Art der Antikoagulationstherapie. Die zwischen 2008 und 2015 zugelassenen oralen Antikoagulationen vom Typ der direkten Faktor-Xa-Inhibierung wie Rivaroxaban, Apixaban oder Edoxaban bzw. der Faktor-IIa-Inhibierung wie Dabigatran ermöglichen eine einfachere Steuerung im Rahmen elektiver Eingriffe. Es ist also anzunehmen, dass auch in der analysierten Kohorte diese Medikation die klassischen oralen Vitamin-K-Antagonisten zurückgedrängt hat.

Die vorliegende Auswertung zeigte im Langzeitverlauf ebenfalls eine signifikante Reduktion der permanenten Stimmbandparese bei der Operation benigner Schilddrüsenerkrankungen. Der nahezu flächendeckende Einsatz des intraoperativen Neuromonitorings mit über 95 % zeigte sich auch in dieser Analyse. Dies korreliert mit Registerdaten des National Surgical Quality Improvement Program (NSQIP) aus den USA, die im 6‑Jahres-Verlauf ein sinkendes Recurrenspareserisiko bei zunehmendem Einsatz des Neuromonitorings ergaben [32].

Während höheres Lebensalter, Rezidivoperation und auch die Thyreoiditis ein signifikant erhöhtes Risiko für die permanente Stimmbandlähmung ergeben, ist bei solitären Schilddrüsenknoten ein signifikant vermindertes Risiko zu verzeichnen (OR 0,53 [0,45–0,63]). Eine Korrelation der Operationsindikation zur Komplikationsrate ist für die unterschiedlichen benignen Schilddrüsenerkrankungen kaum belegt. Das erhöhte Komplikationsrisiko für Rezidiveingriffe ist gut belegt. So zeigt eine rezente Publikation von Aslan et al. eine erhöhte Komplikationsrate für Rezidiveingriffe nach subtotaler Resektion (36,5 %) im Vergleich zu komplettierender Thyreoidektomie nach Lobektomie (11 %) (*p* = 0,04). Und in dieser Studie zeigte sich auch ein Zusammenhang zwischen Knotengröße und Komplikationsrate (*p* = 0,03) [33]. Eine weitere kürzlich publizierte multizentrische Analyse bestätigte einen Zusammenhang zwischen Ausmaß der Resektion und Recurrenspareserate (1,7 % bei Hemithyreoidektomie und 1,0 % bei Thyreoidektomie) und den Einfluss der Indikation mit erhöhtem Risiko für maligne Erkrankungen (OR 4,00; 1,37–11,64) und diffuse Struma (OR 4,94; 0,86–28,37) [34].

Trotz der weiten Verbreitung des intraoperativen Neuromonitorings in Deutschland ergibt sich besonders für die Stimmbandpareserate der Zusammenhang zwischen Fallzahl und Komplikationsrisiko. Die Analyse bestätigte einen signifikanten Unterschied zwischen den fallzahlstärksten Krankenhauskategorien im Vergleich zu fallzahlschwachen Kliniken. Die oben genannte StudoQ-Auswertung und auch vorhergehende Veröffentlichen aus AOK-Routinedaten bestätigten diesen Trend [19, 20, 32].

Die Korrelation zwischen Fallzahl und Ergebnisqualität wird auch im Zuge der gesundheitspolitischen Diskussion an Gewicht gewinnen. In einigen Bereichen konnte bereits eine klare Assoziation zwischen Krankenhausvolumen bzw. Fallzahl und Ergebnisqualität für deutsche Kliniken gezeigt werden. So untersuchten Nimptsch und Mansky 2017 verschiedene viszeralchirurgische Eingriffe [35] und Hentschker und Menniken 2018 Hüftfrakturen [36]. Beide Studien bezogen sich allerdings auf die mit wachsender Fallzahl sinkende Mortalität.

Die Mortalität ist jedoch kein geeigneter Parameter für die Untersuchung der Behandlungsqualität von Schilddrüseneingriffen. Liu et al. untersuchten die typischen Komplikationen der Schilddrüsenchirurgie auf ihre Eignung als Qualitätsparameter für Kliniken in den USA [37]. Sie konnten zeigen, dass v. a. für Hypokalzämie und Stimmbandparese ein Qualitätsunterschied der Kliniken existierte, wobei sich v. a. Unterschiede in der prozeduralen Qualität, z. B. bei der Verwendung des intraoperativen Neuromonitorings, ergaben. Einen signifikanten Einfluss des intraoperativen Neuromonitorings zeigte sich in der vorliegenden Studie nicht. Somit bleibt doch der Verweis auf die „Practice makes perfect“-Theorie [35]. Die Hypokalzämie als Komplikationsparameter ist in den Routinedaten der AOK nicht ausreichend zu analysieren.

Die in dieser Studie beobachtete Verschiebung der Fallverteilung hin zu fallzahlstarken Kliniken ging mit einer gleichzeitigen allgemeinen Fallzahlreduktion einher. Die Untersuchung zeigte, dass diese Verschiebung nicht den beobachteten Rückgang der Komplikationen erklärte. Dennoch konnte für die permanenten Stimmbandparesen ein klarer Volume-Outcome Zusammenhang gezeigt werden. Da auch im Jahr 2023 gemäß den Angaben der strukturierten Qualitätsberichte immer noch über 240 Kliniken deutschlandweit nicht mehr als 5 Schilddrüseneingriffe durchführten, ist das Potenzial zur weiteren Reduktion von Komplikationen vermutlich noch nicht gehoben. Auch Minz et al. zeigen im Allgemeinen eine große Anzahl von Kliniken, die weniger als 30 Fälle einer Erkrankung pro Jahr im Durchschnitt behandeln, und postulieren eine wünschenswerte Spezialisierung zur Steigerung der Qualität [38].

## Limitationen

Diese Studie unterliegt verschiedenen Limitationen. Erstens handelt es sich um eine Sekundärdatenanalyse auf Basis von Routinedaten. Es konnten nur Patient:inneneigenschaften und Komplikationsereignisse betrachtet werden, die anhand der zur Verfügung stehenden Katalogsysteme valide abgebildet werden konnten. Eine Unter- oder Überdokumentation einzelner Ereignisse ist nicht auszuschließen. Weiterhin ist es möglich, dass die Häufigkeit der Stimmlippenlähmungen unterschätzt wurde, da entsprechend der Leitlinienempfehlung nur Patient:innen berücksichtigt wurden, bei denen eine Lupenlaryngoskopie durchgeführt wurde [39].

Für die Untersuchung der Volume-Outcome-Zusammenhänge stand ausschließlich die jährliche Anzahl an Schilddrüsenoperationen je Krankenhaus zur Verfügung. Die Anzahl an Operationsteams, auf die sich dieses Fallvolumen verteilt, wurde nicht berücksichtigt. Es ist davon auszugehen, dass in vielen Krankenhäusern mehrere Operationsteams arbeiten. In diesem Fall wäre das Fallvolumen der Operationsteams niedriger als das gesamte Fallvolumen des Krankenhauses.

Eine weitere Limitation ist, dass der Endpunkt Hypokalzämie nicht untersucht werden konnte, da weder die Auswertung stationärer oder ambulanter Daten noch die Rezeptierung von Kalzium- oder Vitamin-D-Produkten eine Schlussfolgerung auf das Vorliegen einer Hypokalzämie erlaubten. Eine Erweiterung der Analysen um diesen Endpunkt wäre wünschenswert, da er häufiger als die postoperative permanente Stimmlippenlähmung auftritt und ebenfalls die Lebensqualität beeinflusst.

Mit Blick auf die Generalisierbarkeit bestehen Einschränkungen, da das betrachtete Patient:innenkollektiv ausschließlich aus AOK-Versicherten besteht. Obwohl das Versichertenkollektiv der AOK ca. ein Drittel aller Krankenhausfälle in Deutschland darstellt, bestehen gewisse Unterschiede zur Versichertenpopulation anderer Krankenkassen hinsichtlich Altersstruktur und Komorbidität [40].

## Fazit

- Zusammengefasst zeigt sich anhand der vorliegenden Daten eine Abnahme der Eingriffshäufigkeit bei benignen Schilddrüsenerkrankungen im 10-Jahres-Verlauf in Deutschland, wobei eine Verschiebung der Eingriffshäufigkeit hin zu fallzahlstarken Kliniken stattfindet.
- Zudem lässt sich eine signifikante Verbesserung der Komplikationsraten für permanente Stimmbandparese und revisionsbedürftige Nachblutung zeigen. Dieser Rückgang an Komplikationen konnte weder durch patient:innenspezifische Risikofaktoren noch durch die beobachtete Verschiebung der Fallverteilung erklärt werden.
- Eine Konzentration von Schilddrüseneingriffen in spezialisierten Kliniken scheint auch in Zukunft zugunsten der Patientinnen und Patienten wünschenswert. Gerade im Rahmen elektiver Eingriffe wie der Schilddrüsenchirurgie benigner Erkrankungen sollte auch weiterhin die Behandlungsqualität im Vordergrund stehen.

## Supplementary Information

Zusätzliche Tab. 5: Logistische Regressionsanalyse zur Bestimmung des Einflusses (Odds Ratio) der Zeit auf die Endpunkte unter Berücksichtigung weiterer Risikofaktoren (AOK-Fälle 2011–2021)

## Data Availability

Die in dieser Studie verwendeten Daten befinden sich auf einem sicheren Datenspeicher im Wissenschaftlichen Institut der AOK. Der Zugang zu Daten der gesetzlichen Krankenkassen für Forschungszwecke ist nur unter den im Sozialgesetzbuch (SGB § 287) festgelegten Bedingungen möglich. Anträge auf Datenzugang können beim korrespondierenden Autor eingereicht werden.
