# Supplementary material for: Operations for benign thyroid gland diseases in Germany—Development of case numbers and complication rates over the course of 10 years
Source: Chirurgie (Heidelb). 2026 Jan 30;97(8):655–65. [Article in German] doi: 10.1007/s00104-026-02459-4 (PMC13421284; doi:10.1007/s00104-026-02459-4)
Supplement: Supplementary file 1 — Zusätzliche Tab. 5: Logistische Regressionsanalyse zur Bestimmung des Einflusses (Odds Ratio) der Zeit auf die Endpunkte unter Berücksichtigung weiterer Risikofaktoren (AOK-Fälle 2011–2021) [file 104_2026_2459_MOESM1_ESM.pdf]

Tabelle 5: Logistische Regressionsanalyse zur Bestimmung des Einflusses (Odds Ratio) der Zeit auf die Endpunkte unter Berücksichtigung weiterer Risikofaktoren (AOK-Fälle 2011-2021)

	Permanente Stimmbandlähmung	Logopädie (≥21 Einheiten)	Revisionsbedürftige Blutung	Revisionsbedürftige Wundinfektion
**Zeit in Jahren**	0,97 (0,95-0,98)	0,98 (0,97-1,01)	0,96 (0,95-0,98)	0,97 (0,94-1,01)
**Alter in Jahren**				
18-42	1 (Referenz)	1 (Referenz)	1 (Referenz)	1 (Referenz)
43-50	1,27 (1,07-1,51)	1,47 (1,20-1,80)	1,25 (1,08-1,45)	-
51-57	1,50 (1,29-1,74)	1,67 (1,35-2,07)	1,63 (1,42-1,88)	-
58-66	1,90 (1,62-2,21)	1,81 (1,48-2,23)	1,74 (1,50-2,01)	-
≥67	2,42 (2,09-2,80)	2,13 (1,73-2,62)	1,67 (1,43-1,94)	-
**Geschlecht (w)**	1,23 (1,10-1,37)	1,62 (1,38-1,89)	0,73 (0,67-0,80)	0,36 (0,30-0,43)
**OP-Verfahren und Morphologie**				
Reexploration	2,21 (1,79-2,73)	1,98 (1,56-2,51)	1,40 (1,10-1,79)	-
Beidseitige Operation	0,69 (0,62-0,77)	0,76 (0,66-0,88)	1,19 (1,08-1,32)	-
Mehrknotige Struma	-	-	1,28 (1,14-1,43)	-
Solitärer Schilddrüsenknoten	0,54 (0,45-0,64)	0,52 (0,42-0,65)	-	-
Thyreoiditis	1,51 (1,12-2,05)	-	-	-
Basedow-Krankheit [b]	-	0,68 (0,51-0,91)	1,51 (1,25-1,82)	-
Neuromonitoring	0,88 (0,74-1,04)	0,82 (0,66-1,01)	-	-
**präoperative Medikation**				
systemische Glukokortikoide	-	-	0,74 (0,58-0,95)	-
Thyreostatika	-	-	1,37 (1,22-1,53)	-
**Begleiterkrankungen**				
Störungen des Flüssigkeits- und Elektrolythaushalts	1,69 (1,38-2,07)	1,89 (1,41-2,54)	2,69 (2,25-3,22)	3,28 (2,46-4,37)
Kardiale Arrhythmie	-	-	1,62 (1,39-1,89)	1,47 (1,08-2,01)
Koagulopathie	-	-	4,07 (3,29-5,04)	2,45 (1,49-4,03)
Adipositas	-	-	0,83 (0,74-0,93)	1,78 (1,40-2,27)
Alkoholabusus	-	-	2,19 (1,29-3,74)	-
Bluthochdruck, ohne Komplikationen	-	-	1,15 (1,06-1,25)	-
Weitere neurologische Erkrankungen	-	-	1,39 (1,04-1,85)	-
Diabetes, ohne Komplikationen	-	-	-	1,71 (1,37-2,12)
Gewichtsverlust	-	-	-	6,37 (3,27-12,4)
Chronische Lungenerkrankung	1,36 (1,16-1,60)	-	-	-
Lähmung	1,83 (1,04-3,20)	-	-	-
Defizienzanämie	-	2,27 (1,08-4,79)	-	-
Depression	-	1,38 (1,02-1,85)	-	-

Mit „–“ gekennzeichnete Risikofaktoren waren aufgrund fehlender Signifikanz nicht in der Risikoadjustierung enthalten.

[b] ICD-10 E05.0 Hyperthyreose mit diffuser Struma (Inklusion: Basedow-Krankheit, toxische diffuse Struma, toxische Struma ohne nähere Angabe)
